# Robust expansion and functional maturation of human hepatoblasts by chemical strategy

**DOI:** 10.1186/s13287-021-02233-9

**Published:** 2021-02-25

**Authors:** Tingcai Pan, Jiawang Tao, Yan Chen, Jiaye Zhang, Anteneh Getachew, Yuanqi Zhuang, Ning Wang, Yingying Xu, Shenglin Tan, Ji Fang, Fan Yang, Xianhua Lin, Kai You, Yi Gao, Yin-xiong Li

**Affiliations:** 1 Institute of Public Health, Guangzhou Institutes of Biomedicine and Health (GIBH), Chinese Academy of Sciences, Guangzhou, 510530 China; 2 Key Laboratory of Regenerative Biology, South China Institute for Stem Cell Biology and Regenerative Medicine, Guangdong Provincial Key Laboratory of Biocomputing, Guangzhou Institutes of Biomedicine and Health, Chinese Academy of Sciences, Guangzhou, 510530 China; 3 Department of Hepatobiliary Surgery II, Zhujiang Hospital, Southern Medical University, Guangzhou, 510280 Guangdong Province China; 4 University of Chinese Academy of Science, Beijing, 100049 China; 5 Bioland Laboratory (Guangzhou Regenerative Medicine and Health Guangdong Laboratory), Guangzhou, 510005 China

**Keywords:** Hepatoblast expansion, Hepatic maturation, Stemness maintenance, Small molecules, Chemical cocktail

## Abstract

**Background:**

Chemically strategies to generate hepatic cells from human pluripotent stem cells (hPSCs) for the potential clinical application have been improved. However, producing high quality and large quantities of hepatic cells remain challenging, especially in terms of step-wise efficacy and cost-effective production requires more improvements.

**Methods:**

Here, we systematically evaluated chemical compounds for hepatoblast (HB) expansion and maturation to establish a robust, cost-effective, and reproducible methodology for self-renewal HBs and functional hepatocyte-like cell (HLC) production.

**Results:**

The established chemical cocktail could enable HBs to proliferate nearly 3000 folds within 3 weeks with preserved bipotency. Moreover, those expanded HBs could be further efficiently differentiated into homogenous HLCs which displayed typical morphologic features and functionality as mature hepatocytes including hepatocyte identity marker expression and key functional activities such as cytochrome P450 metabolism activities and urea secretion. Importantly, the transplanted HBs in the injured liver of immune-defect mice differentiated as hepatocytes, engraft, and repopulate in the injured loci of the recipient liver.

**Conclusion:**

Together, this chemical compound-based HLC generation method presents an efficient and cost-effective platform for the large-scale production of functional human hepatic cells for cell-based therapy and drug discovery application.

**Supplementary Information:**

The online version contains supplementary material available at 10.1186/s13287-021-02233-9.

## Background

A robust and cost-effective expansion and differentiation method for large-scale production of functional human hepatocytes could be beneficial for many potential clinical applications, including hepatic cell transplantation, bio-artificial liver, drug development, and disease modeling [1, 2]. To date, protocols have been developed to generate hepatoblasts (HBs) and hepatocyte-like cells (HLCs) from human pluripotent stem cells (hPSCs) in vitro [3–5]. Despite improvements in differentiation efficiency and functional maturation have been achieved, while current protocols are unsatisfying both quantitatively (limited amount of the hPSCs can be directly induced to mature) and safety (undifferentiated hPSCs may remain). Hence, the technical challenges relate to scalability, reproducibility, and cost-effective need to be solved for the production of sufficient quality and quantify functional hepatocytes to meet clinical and another widespread applications.

HBs are capable of self-renewal and have the potency to differentiate into hepatocytes and hence expand purified HBs derived from hPSCs which would be an ideal strategy to enable massive hepatic cell production. Our previous study has established a cocktail (ABCEHS) to synergistically regulate BMP, Wnt, Hedgehog, and other signaling pathways that created a fine balance for HB expansion and bipotency maintenance [6]. Recent studies also reported hepatic expansion and maturation methods mostly applied expensive growth factors, which leads to critical challenges such as the high cost and protocol reproducibility in large-scale production [7–9]. As an alternative, chemical compounds cost less and more stable than growth factors. This suggested a cost-effective and reproducible approach by using chemical compounds for producing scalable and integrated functional hepatocytes.

In the present study, we focused on improving efficient and cost-effective hepatic expansion and maturation conditions, consisting of small molecules and chemical define culture systems. We first established an optimal chemical culture cocktail ACDFSV (A8301, CHIR99021, Dihexa, FSK, SAG, and vitamin C) for HB expansion and bipotency maintenance. Then, the transition of HBs into HLCs was induced by chemical cocktail DNAD (Dex, NH4Cl, A8301, and Dihexa). The chemically derived HLCs showed typical morphologic and functional features of mature hepatocytes both in vitro and in vivo. In summary, our current chemical-based hepatic expansion and maturation method presents an efficient and robust platform for the large-scale production of functional human hepatocytes. This approach showed a promising future in providing cost-effective and qualified hepatocytes for clinical regenerative medicine and drug discovery applications and a solid foundation for building bioreactors for the bio-artificial liver.

## Methods

### Human HB culture and hepatic maturation

HBs were generated from hPSCs and maintained in expansion medium as described previously [6]. Briefly, the hPSCs were differentiated into HB and the Ep-CAM^+^/C-Kit^−^ cells were purified by FACS from differentiation cultures and maintained on the growth factor reduced Matrigel (Corning).

**Fig. 1:**
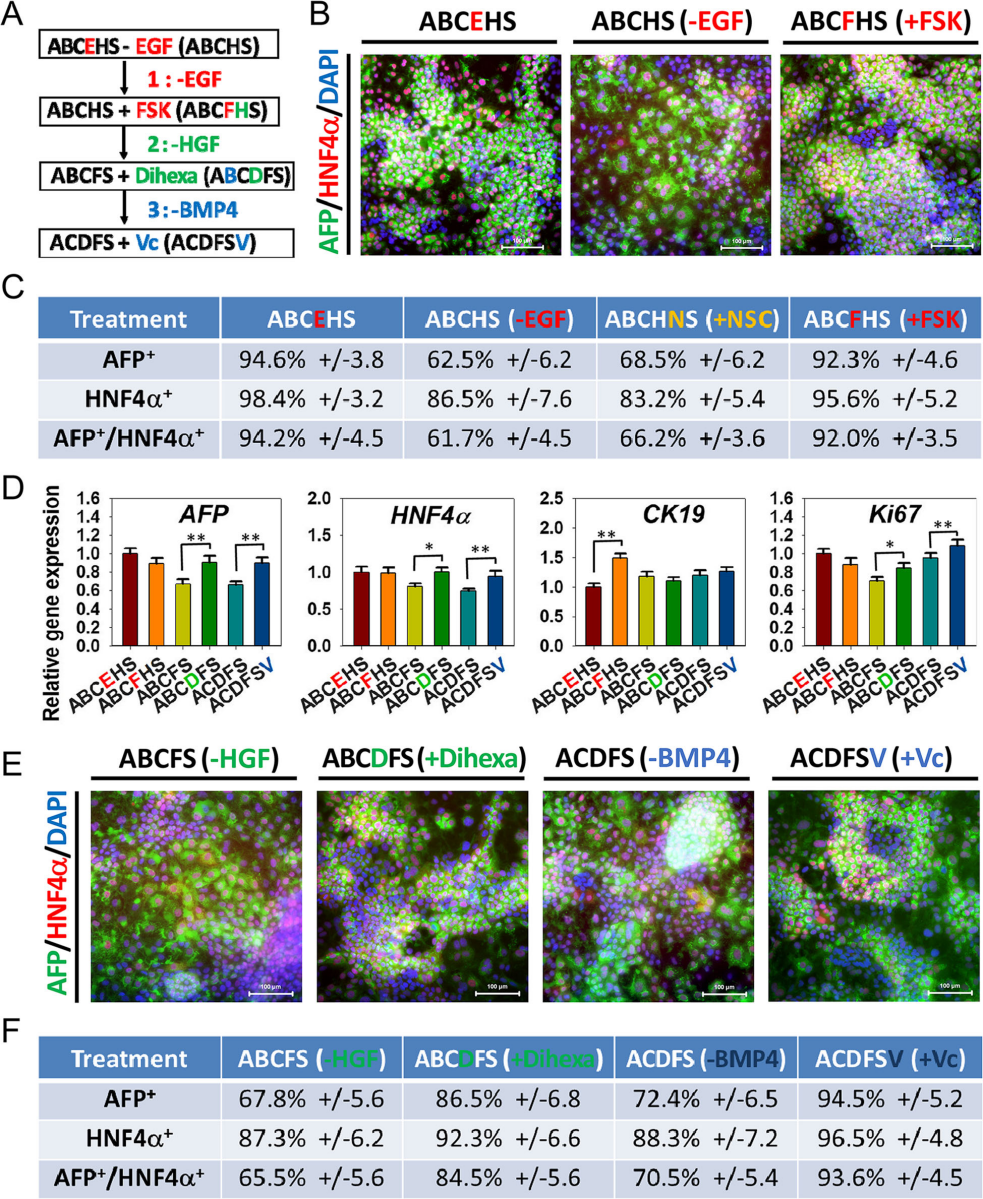
Establishment of a chemical culture cocktail for HB expansion. **a** Schematic of the strategy to identify chemical culture cocktail for HB expansion. **b** Immunostaining analyses of HB-specific marker AFP and HNF4α expression after different chemical cocktails treated. Scale bars 100 μm. **c** Efficiency of AFP and HNF4α expression after different chemical cocktails treatment, determined by counting positive cells. Efficiencies are presented as the percentage of positive cells plus or minus the SD of all fields counted. **d** Quantitative RT-PCR analyzed hepatic genes and *Ki67* expression in chemical cocktails treated HBs. Data are presented as mean ± SEM, *n* = 3. *P < 0.05, **P < 0.01. **e** Immunostaining analyses of AFP and HNF4α expression in chemical cocktails treated HBs. Scale bars 100 μm. **f** Efficiency of AFP and HNF4α expression after different chemical cocktails treatment, determined by counting positive cells. Efficiencies are presented as the percentage of positive cells plus or minus the SD of all fields counted

**Fig. 2:**
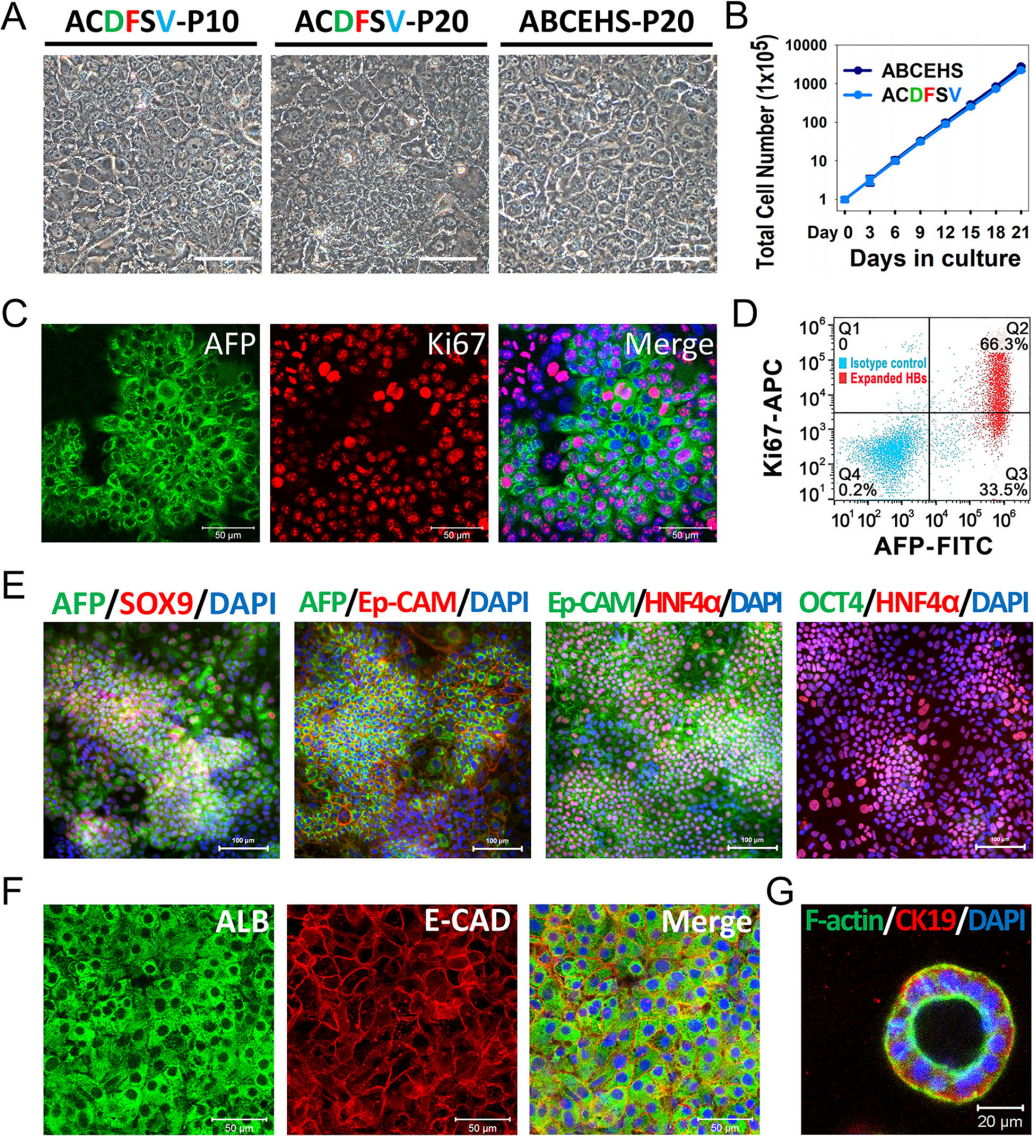
The ACDFSV culture condition enables long-term self-renewal and maintenance of HBs. **a** Phase-contrast images of expanding HBs. Scale bar 100 μm. **b** Growth curve of HBs cultured in ABCEHS cocktail and ACDFSV cocktail conditions. Cell growth curves were analyzed by obtaining a cell count. Data are presented as mean ± SEM, *n* = 3. **c** Immunostaining analyses of AFP and Ki67 expression on expanded HBs (passage 10). Scale bars 50 μm. **d** Flow cytometric analyses of AFP and Ki67 expression in expanded HBs (passage 10). **e** Immunostaining analyses of HB marker proteins on expanded HBs (passage 10). Scale bars 100 μm. **f** Expanded HBs differentiated into ALB and E-CAD co-staining hepatocytes after mature induction. Scale bars 50 μm. **g** Expanded HBs differentiated into CK19 and F-actin co-staining bile duct-like structures in 3D culture. Scale bar 20 μm

For HB chemical cocktail expansion, the purified HBs were cultured in expansion basal medium (RPMI1640, 1× B27 supplement, 1× ITS [insulin-transferrin-sodium selenite, Sigma-Aldrich]) supplemented with chemical cocktails as indicated in Fig. 1a. Chemical compounds were tested at appropriate concentrations as routinely used, including 5 μM A8301, 3 μM CHIR99021, 0.1 μM Dihexa, 10 μM Forskolin (FSK, Stemgent), 50 μM NSC228155, 0.5 μM SAG, and 10 μg/mL vitamin C (Vc, Sigma-Aldrich) (Fig. 2). The chemical compounds used were purchased from Selleck except indicated. For cell replating in our current method, the expanding HBs were dissociated by Accutase (life) and replated on Matrigel Matrix pre-coated plates.

**Fig. 3:**
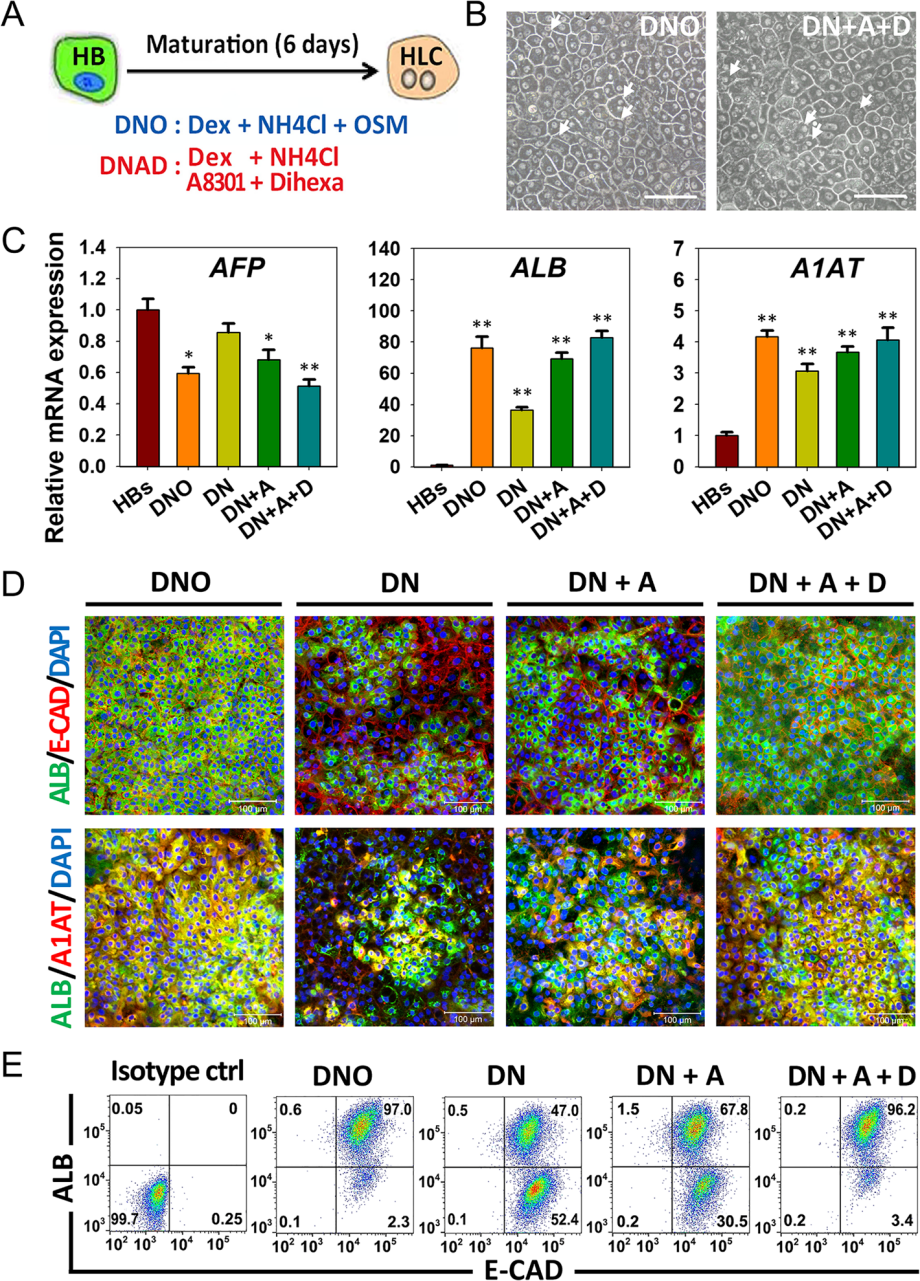
Establishment of a chemical induction cocktail for HLC maturation. **a** Schematic of screening induction condition for hepatic maturation. **b** The morphology of expanded HB-derived HLCs. HLCs exhibited distinct round nuclei even some has two nuclei (white arrows). Scale bar 100 μm. **c** Quantitative RT-PCR analyzed *AFP*, *ALB*, and *A1AT* expression in HLCs derived from different induction cocktails. Gene expression was normalized to HBs. Data are presented as mean ± SEM, *n* = 3. *P < 0.05, **P < 0.01. **d** Immunostaining analyses of hepatocyte markers ALB, E-CAD, and A1AT on HLCs derived from different induction cocktails. Scale bars 100 μm. **e** Flow cytometric analyses for ALB and E-CAD positive rate on HLCs derived from different induction cocktails

For hepatocyte maturation, HBs were cultured in hepatoZYME-SFM (Gibco) medium supply with 1X GlutaMAX, containing small molecules as indicated in Fig. 3a, including 5 μM A8301, 0.1 μM dexamethasone (Dex; Sigma-Aldrich), 0.1 μM Dihexa, and 0.5 mM NH4Cl (Sigma) for 6 days. The medium was changed daily during the differentiation period. The small molecules used were purchase from Selleck, and growth factors were purchased from PeproTech unless otherwise indicated.

For bile duct induction, dissociated HBs were suspended in basal medium (RPMI1640 [Gibco], supply with 1 × B27 [Invitrogen]) supplemented with 20 ng/mL EGF and 20 ng/mL HGF and mixed 1:1 with Matrigel. Then, the mixture was plated into 24-well plates (0.5 mL/well) and placed in an incubator at 37 °C for 2 h to allow the formation of 3D Matrix. The cells were cultured for approximately 1 week to allow the formation of bile duct-like structures. The medium was changed carefully every other day.

### Functional analyses of differentiated HLCs in vitro

For the urea secretion assay, the culture media of 24 h incubated in differentiated cells were collected and stored at − 80 °C. Urea concentration was analyzed by LC/MS/MS API3000 and normalized with total cell protein concentration.

To evaluate the CYP450 activity, cultures were incubated with conventional probe substrates (CYP3A4: 6 μM midazolam, CYP2C9: 10 μM diclofenan, CYP2D6: 10 μM dextromethorphan) respectively, for quantifying metabolite production. After 2 h exposure, culture medium was collected and stored at − 80 °C subsequently, and LC/MS/MS API3000 analyzed CYP450 activity. Metabolite products were normalized to total cell protein.

To evaluate the glycogen production and storage ability, periodic acid-Schiff (PAS) staining was performed. According to the manufacturer’s instructions, the cultured cells were fixed with 4% PFA for 30 min, and intracellular glycogen was stained using a PAS staining solution (Muto Pure Chemicals).

For cellular indocyanine green (ICG) uptake assay, differentiated HLCs were incubated in media supplemented with ICG (final concentration of 1 mg/ml, Sigma-Aldrich) for 1 h at 37 °C. Cells were then washed with PBS and imaged using phase contrast microscope (X71, Olympus).

### Animal model and hepatic cells transplantation

Immune-deficient NOD-SCID-IL2RG^−/−^ mice (NSI mice, the reference number of institutional animal care and use committee: 2018010, GIBH) were used as recipients of human hepatic cells. Before hepatic cells transplantation, 8-week-old NSI mice received dimethylnitrosamine (DMN) intraperitoneal injections (7 mg/kg, Sigma, 1.0% dissolved in saline) for 2 consecutive days for inducing acute liver injury. Two days later, 1 × 10^6^ hepatic cells were intrasplenically transplanted into the DMN-treated NSI mice. To monitor the transplantation state, recipient mice livers were harvested at different time points after hepatic cells transplantation. Additionally, human hepatocytes producing the ALB protein were identified in mice liver by an antibody specifically recognizing human ALB. All animal experiments were approved by the Animal Welfare Committee of GIBH. All protocols were approved by the relevant institutional animal care and use committee (IACUC).

### Immunohistochemistry staining

Cells were washed with PBS and fixed with 4% PFA (Sigma-Aldrich) for 30 min at room temperature. After washing the cells with PBS, cells were permeabilized with 0.1% Triton X-100 in PBS for 30 min and blocked with PBS containing 5% normal goat or donkey serum for 30 min at room temperature. The primary antibodies were diluted with blocking solution and incubated at 4 °C overnight. After washing the cells with PBS, cells were then stained with compatible Alexa Fluor-conjugated secondary antibodies in blocking solution for 1 h at room temperature. The nucleus was stained with 5 μg/mL DAPI (Invitrogen). Imaging was performed on a Zeiss LSM 710 confocal microscope. The primary antibodies and secondary antibodies are described in the Supporting information Table 1.

### Quantitative RT-PCR and microarray analysis

Total RNA was extracted using TRIzol reagent (Invitrogen) according to the manufacturer’s protocol and quantified with NanoDrop 2000 (Thermo Fisher). The cDNAs were reverse transcribed from 2 μg RNA using ReverTra Ace (Toyobo) and oligo-dT (Takara). Quantitative RT-PCR was performed with CFX96 machine (Bio-Rad) and SYBR Green Premix (Bio-Rad) following the manufactures’ manual. The GAPDH was used for quantitative RT-PCR normalization, and the experiments were repeated a minimum of three times to confirm the results. Primer sequences are listed in the Supporting information Table 2.

### Statistical analysis

The data were analyzed with Sigma Plot 10.0 Statistical differences between two groups were tested with a two-tailed Student’s *t* test. Data is represented as mean ± SEM. Survival data were analyzed with the Kaplan-Meier test. For all tests, *p < 0.05 was considered significant.

## Results

### Establishment of a chemical culture cocktail for HB expansion

To replace the growth factors (BMP, EGF, and HGF) in the previous established cocktail (ABCEHS) with small molecules for developing a full chemical strategy for HB expansion, we performed a sequential screening procedure to screen small molecules targeting the relevant signaling pathways to identify an ideal combination of cocktail for the proliferation of HBs and maintain their bipotency (Fig. 1a).

Consistent with previous results, HBs gave rise to rapid proliferation and stemness maintenance upon ABCEHS treatment, while withdrawn EGF or another cytokine individually, the cell proliferation was only decayed slightly (Figure S1). Thus, we focus on the outcomes of hepatic characteristic maintenance during the small molecule screening process. Withdrawal of EGF led to decreasing expression of *AFP*, *CK19*, and cell proliferation marker *Ki67* (Figure S2). However, chemically activating EGF receptor with NSC228155 (NSC) could not restore HB culture and genes expression (Figure S2). To overcome this issue, we screened small molecules that can activate EGF relative downstream pathways (ERK, Akt, and PKA) in EGF absent conditional media (ABCHS). The results showed that forskolin (FSK), an adenylate cyclase activator and thus activating PKA signal transduction, could replace EGF to support HB culture (Figure S1) and restore genes expression (Figure S2). Moreover, immunostaining with AFP and HNF4α expression further confirmed the function of FSK in HB stemness maintenance (Figs. 1b, c, S3).

To further replace HGF, we found that Dihexa, a HGF agonist, could mimic the function of HGF in HB expansion and maintenance (Fig. 1d, e, f). However, in the last step to replace BMP4, we encountered difficulties due to the lack of reliable agonist in BMP pathway. Fortunately, we found that Vc, which had been reported that could phosphorylate SMAD1/5/8 and potentiate BMP signaling pathway activity in cardiomyogenesis [10], could phosphorylate SMAD1/5/8 in HB culture (Figure S4). As expected, Vc administration improved cell growth (Figure S1), and the expression of hepatic genes *AFP* and *HNF4*α were also restored effectively (Fig. 1d). These results were further confirmed by immunostaining with HB biomarkers, displaying strong AFP and HNF4α co-staining by Vc addition (Fig. 1e, f).

### The ACDFSV culture condition enables long-term self-renewal and maintenance of HBs

To further address whether chemical cocktail (ACDFSV) could support HB expansion for long-term culture without diminishing their bipotency, we cultured HB from a single cell in ACDFSV-treated condition for at least 20 passages. The result showed that single-cell HBs were able to proliferate and form colonies in several days (data not showed) and maintained a similar morphology between early (P10) and midterm (P20) passages (Fig. 2a). Additionally, the number of proliferative HBs grew nearly 3000 times within 3 weeks without obvious proliferation decay (Fig. 2b).

For HB characterization, immunostaining was performed and revealed the majority of expanded cells were AFP positive, and a considerable number of AFP positive cells co-expressed Ki67 (Figs. 2c, S5). Flow cytometry result further confirmed that 66.3% AFP-positive HBs co-expressed Ki67, indicating preferable proliferative ability of expanded HBs (at P20) (Fig. 2d). Further results in the proliferative HBs exhibited features of bipotency, with co-expression of the hepatic AFP, HNF4α, and the early cholangiocyte marker SOX9 and Ep-CAM. Importantly, these cells were negative for pluripotent OCT4 (Fig. 2e).

To evaluate the differentiation capacity of expanded HBs, we then cultured the HBs in the previously established hepatic maturation condition Dex + NH4Cl + OSM (DNO), and immunostaining was performed after 6 days induction. The results revealed that hepatocyte-specific proteins ALB and E-CAD were strongly co-expressed in the derived hepatocytes (Fig. 2f). In addition, HBs could differentiate into cholangiocyte-like cells and form a bile duct-like structure after 1 week in 3D culture. This demonstrated the epithelial polarity with CK19 on the basolateral region and F-actin at the apical region (Fig. 2g).

Taken together, these results indicated that expanded HBs retained bipotency after long-term expansion, with the ability to differentiate into both hepatocytes and biliary lineages in vitro. Moreover, these cells also could be successfully cryopreserved and recovered during expansion (Figure S6). These results demonstrated that the chemical combination ACDFSV is sufficient for the long-term expansion and maintenance of HBs.

### Establishment of a chemical induction cocktail for HLC maturation

Based on previously established hepatic maturation cocktail (DNO), we further optimized chemical condition to induce the expanded HBs differentiate into HLCs, given that the majority of hepatic maturation procedures applied Dex and HGF. Besides, NH4Cl has been demonstrated to promote hepatic genes expression and hepatocyte maturation in previous studies. And recent studies have identified TGF-β inhibitors A8301 or SB431542 both could promote hepatic maturation and functional maintenance of primer hepatocytes (PHs) [8, 11, 12]. Thus, we tried to replace OSM with chemical compound A8301 and HGF mimetic Dihexa, while kept the presence of Dex and NH4Cl in the culture condition (Fig. 3a).

After 6 days of inducing chemical condition, the expanded HBs efficiently differentiated into homogenous HLCs with typical hepatocyte morphology, a polygonal shape and distinct round nuclei. Some of the HLC even have two nuclei, similar to DNO-driven HLCs (Fig. 3b, Figure S7). Further RT-PCR analyses showed that withdrawal OSM resulted in decreased hepatocyte marker genes *ALB* and *A1AT* expression. In contrast, the expression of *AFP* was increased, indicating there might be an insufficient signal for hepatic maturation. In comparison, addition of A8301 results improved expression of *ALB* and *A1AT* as expected. Meanwhile, the inclusion of Dihexa was further increased the expression of hepatocyte makers and decreased the expression of *AFP* (Fig. 3c). Further immunostaining and flow cytometry analyses demonstrated similar results, withdrawing OSM decreased hepatocyte markers ALB and A1AT expression dramatically, while supplying with A8301 and Dihexa restored markers expression (Fig. 3d, e). Therefore, we concluded that the optimal combination is Dex, NH4Cl, A8301, and Dihexa (DNAD).

### Generation of functional HLCs by two-step chemical strategy

**Fig. 4:**
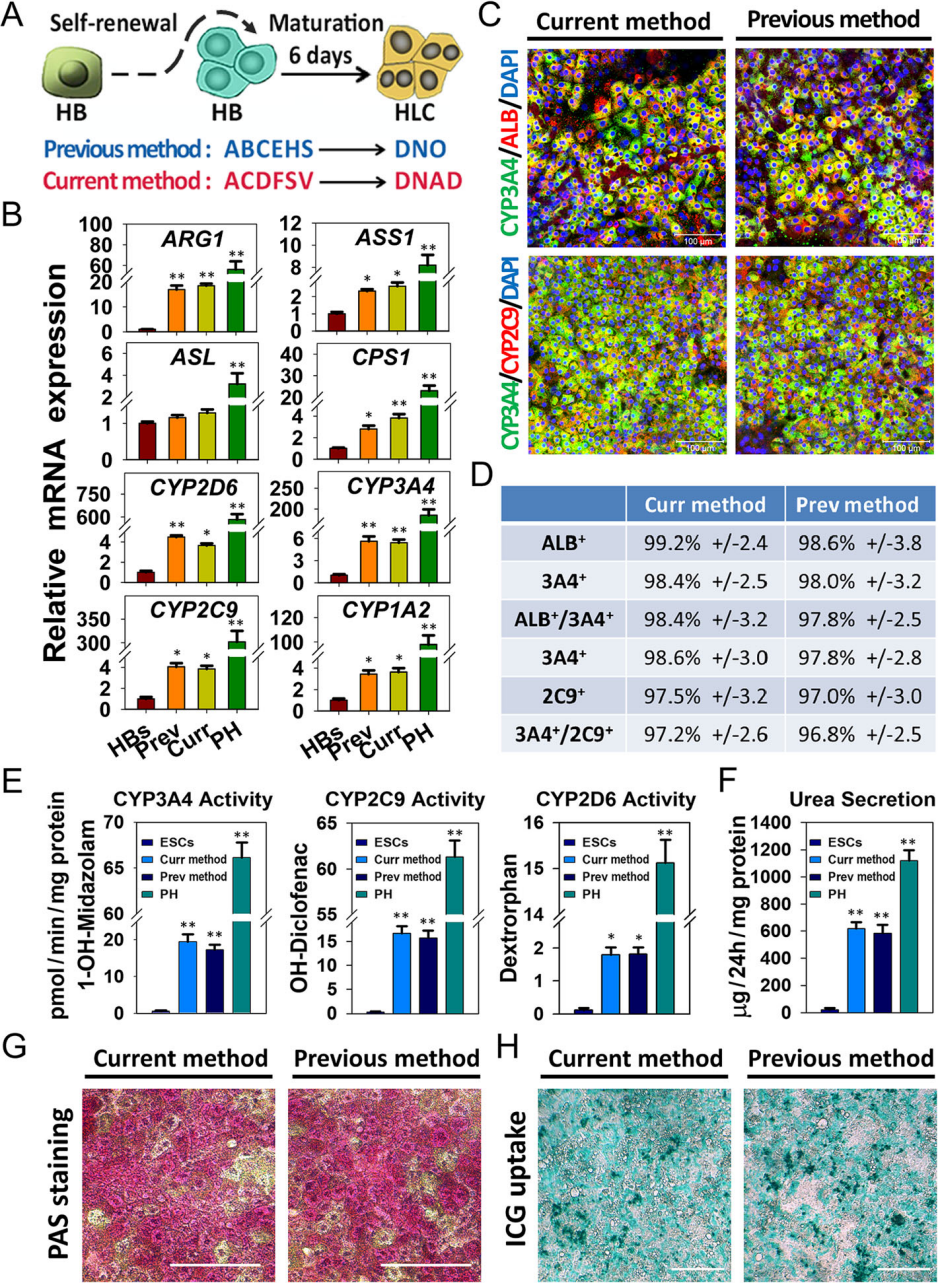
Generation of functional HLCs by two-step chemical strategy. **a** Schematic of the two-step chemical strategy to generate functional HLCs. **b** Quantitative RT-PCR analyzed the genes expression levels of the urea cycle and CYP enzymes. Gene expression was normalized to HBs. Data are presented as mean ± SEM, *n* = 3. *P < 0.05, **P < 0.01. **c** Immunostaining analyses of mature hepatocyte markers (ALB, CYP3A4, and CYP2C9) on HLCs derived from two methods. Scale bars 100 μm. **d** Efficiency of hepatocyte markers expression, determined by counting positive cells. Efficiencies are presented as the percentage of positive cells plus or minus the SD of all fields counted. **e** CYP450 activity assay of different origins of hepatocytes. Data are presented as mean ± SEM, *n* = 3. *P < 0.05, **P < 0.01. **f** Urea secretion among different origins of hepatocytes were analyzed. Data are presented as mean ± SEM, *n* = 3. *P < 0.05, **P < 0.01. **g** PAS staining on different origins of hepatocytes. Scale bar represents 100 μm. **h** ICG uptake analyses in different origins of hepatocytes. Scale bar represents 100 μm

Based on the results above, we established a two-step amplifying culture strategy to generate large quantities of human hepatocytes. Firstly, the hPSC-derived HBs were expanded using ACDFSV expansion medium; then, the medium was changed to the DNAD maturation medium to generate mature hepatocytes (Fig. 4a). To identify the derived HLCs, the expression of hepatic functional relative genes and proteins were analyzed by qRT-PCR and immunostaining. The results indicated that both methods derived HLCs expressed similar urea cycle and cytochrome P450 (CYP) enzymes transcript (Fig. 4b). Moreover, the co-positive of mature hepatocyte-specific proteins ALB, CYP3A4, and CYP2C9 also showed no significant difference in the both methods derived HLCs (Fig. 4c, d).

Further analyses were performed to assess the typical hepatic function of the derived HLCs. We evaluated CYP enzymes’ metabolic activities in derived HLCs, as metabolism and detoxification in the liver are mainly executed by these enzymes in hepatocytes. Three substrates of CYP3A4, 2C9, and 2D6 (Midazolam, Diclofenan, and Dextromethophan, respectively) were used to estimate the drug metabolism capacity of HLCs compared with that of PHs. No significant differences were observed in all three CYP isoforms activity between two methods derived HLCs, although the activity levels were lower than those of PHs (Fig. 4e). These results indicated that the current method-derived HLCs have the capacity to metabolize these substrates. Moreover, HLCs showed a similar urea secretion pattern between the two groups, approximately 50% of the PHs (Fig. 4f).

Additionally, the hepatic functions to store glycogen, uptake indocyanine green (ICG), and ac-LDL and synthesize lipids were also examined. The results exhibited a similar abundant cytoplasmic glycogen storage, ICG and ac-LDL uptake, and lipid synthesizing ability between two groups of HLCs (Figs. 4g, h, S8, S9). Undifferentiated hESCs were used as a negative control in the above experiments. Taken together, the current chemical expansion and maturation method could differentiate HBs into functional mature hepatocytes in a similar highly efficient but more cost-effective than previous method.

### Repopulation of mice injured liver by HB transplantation

**Fig. 5:**
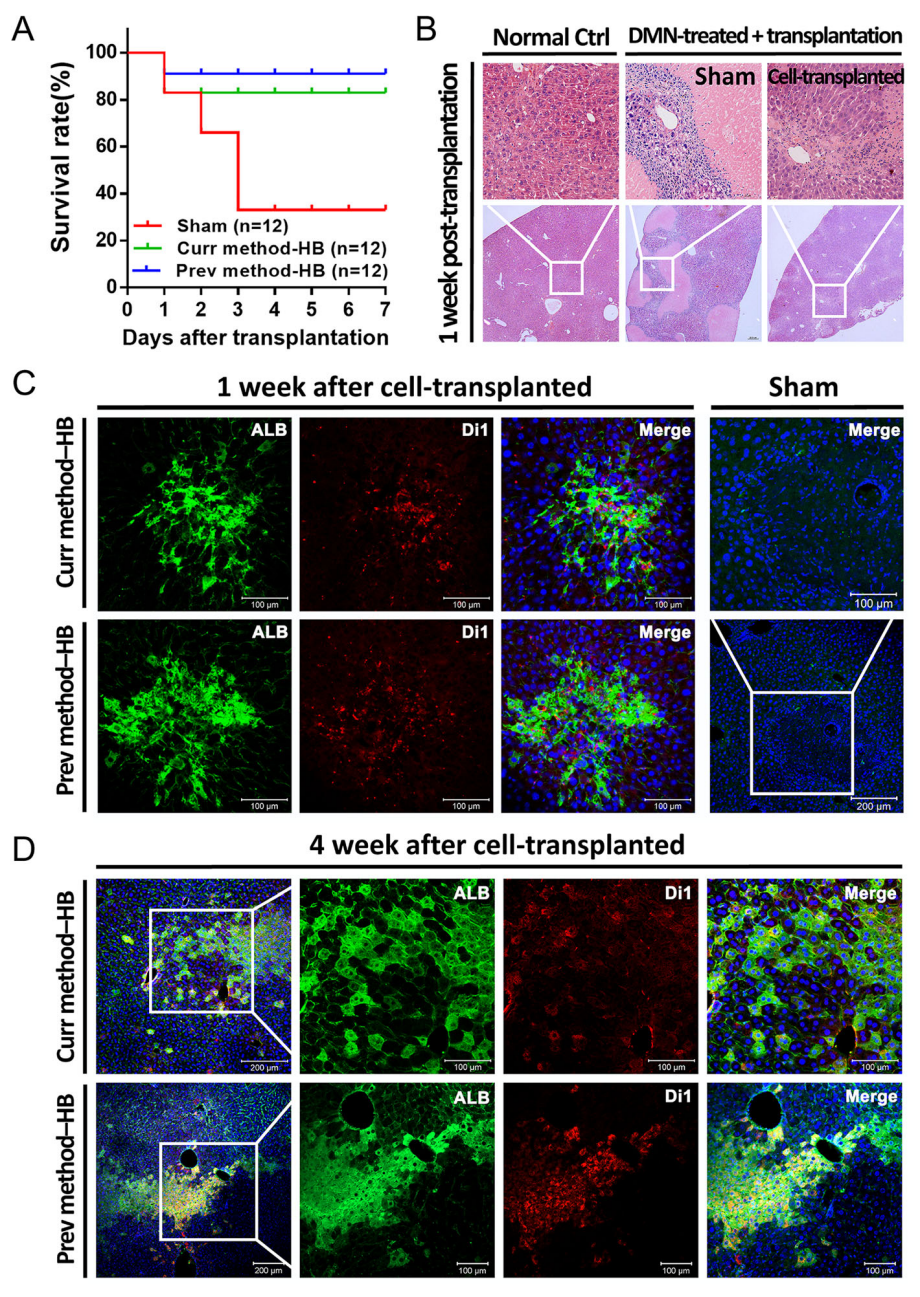
Repopulation of mice injured liver by HB transplantation. **a** Survival curve of mice. **b** Hematoxylin and eosin staining in mice liver. **c** Engraftment of transplanted human HBs in mice liver after 1 week transplantation, as indicated by immunostaining of human ALB (green) and Di1 (red). **d** Repopulation of mice liver after 4 weeks of cell transplantation, as indicated by immunostaining of human ALB (green) and Di1 (red)

To determine whether the expanded HBs could mature into functional hepatocytes in vivo, we transplanted HBs into DMN-induced acute liver failure NSI mice. The Kaplan-Meier survival estimates were determined for 7 days after cell transplantation. In the sham control group, death caused by acute liver failure occurred as early as 3 days and two thirds (8 of 12) died within 5 days after the DMN-injection. For the experimental groups (*n* = 12 per group), both groups’ survival rates were over 80% throughout the examination period (Fig. 5a). Moreover, hematoxylin and eosin (H&E) staining displayed massive necrotic loci in the sham-operated liver sections of DMN-induced acute liver failure (Fig. 5b, second panel). In contrast, the necrotic loci dramatically decreased, and the morphologically nearly restored to normal in the HBs transplanted mice liver (Fig. 5b, first and third panel).

Moreover, to trace the homing cells and analyze the repopulation efficiency, HBs were labeled with Di1 dye before transplantation. After 1 week of transplantation, both Di1 and human ALB positive were found in the HB-transplanted mice liver. In contrast, no human ALB-positive cells were observed in the sham control group (Fig. 5c). These results indicated that HBs derived by both methods could home and differentiate into mature hepatocytes after transplanted in vivo. In addition, the ALT and AST levels in mice serum were significantly reduced after HBs transplantation (Figure S10). At 4 weeks post-transplantation, Di1 and human ALB co-positive cells were observed more widespread in mice liver transplanted with HBs derived by both methods (Fig. 5d). These results demonstrated that the transplanted HBs could engraft, proliferate, and differentiate into hepatocytes and repopulate injured loci in the recipient’s liver.

Moreover, no teratomas or other tumor types were found in any of the transplanted recipients during a 4-week time frame. Therefore, we concluded that the current method derived HBs could engraft and differentiate into functional hepatocytes and repopulate the injured mice liver after transplantation.

## Discussion

Development of efficient and cost-effective technology to generate large quantities and high-quality functional hepatic cells is important for the application of hepatic cells to cell-based therapy and drug toxicity screening. In the present study, we established a robust and cost-effective culture condition for the generation of self-renewing HBs and functional mature HLCs, using a chemical cocktail in a defined culture system. The purified HBs could undergo long-term expansion with preserved bipotency. Furthermore, the expanded HBs could efficiently differentiate into homogenous HLCs displayed typical functional characteristics of adult hepatocytes in vitro and repopulate injured liver parenchyma tissue in vivo.

Recently, it has been reported that PHs can be maintained with five chemicals (5C) [12]. However, this approach faced difficulty in expanding the PHs, compromising the cell quantity. Notably, some efforts have focused on reprogramming PHs to hepatic progenitor-like cells (HPLCs), which retained the ability to re-differentiate into HLCs [13–16]. Although the limited proliferative potential of HPLCs could be improved by HPV E6/E7 or SV40, large T antigen overexpression mediated immortalization to enhance cell proliferation [17–19] and, while under the risk of tumorigenesis, restricts their industrial application.

In contrast, hPSCs-derived HBs show advantages over PHs and reprogramed HPLCs, owing to a wider and more convenient cell source, stronger proliferation ability, and more efficient differentiation into mature hepatocytes [6, 8, 20]. However, previous reported HB amplification formulations contained growth factors that were unsuitable for large-scale cell production. As a solution, chemicals offer an attractive alternative to growth factors since they are more economic-friendly, relatively stable, and possibly more efficient for the generation of hepatic cells. Therefore, replacing growth factors with chemicals and optimizing the hepatic expansion and maturation method, which would be convenient for large-scale and integrated production of functional hepatocytes.

HBs proliferate vigorously and remain bipotent at the liver bud stage during embryogenesis, which is influenced by signals from the surrounding mesenchyme. Similarly, expansion and maintenance of HBs identity in vitro are highly dependent on precise regulation by extrinsic and intrinsic signals [21]. Previous studies have demonstrated that stimulation of Wnt and inhibition of TGF-β were both important for hepatic proliferation [7, 14, 22]. Our recent study correlated with these reports and further demonstrated that Hedgehog signaling is also involved in stemness maintenance and proliferation of HBs [6]. Thus, we kept the TGF-β inhibitor A8301, Wnt agonist CHIR, and Hedgehog activator SAG, while replacing the remaining growth factors (EGF, HGF, and BMP4) with chemicals to optimize the HB culture condition.

We first attempted to screen EGF relative downstream pathways (ERK/MAPK, and cAMP/PKA) agonist for EGF substitution. And we found that FSK could replace EGF to support HB culture and restore hepatic gene expression. Besides, FSK has also been demonstrated essential for liver stem cell expansion and could downregulate EMT marker gene expression and sustain PH gene expression [8, 12, 23]. Furthermore, the chemical culture condition included a potent HGF agonist Dihexa [24], which could mimic the function of HGF in maintaining HBs. Dihexa was identified in a screen that assessed the capacity to potentiate the biological activity of HGF and was originally developed as an anti-dementia drug [25]. Another signaling pathway that needed to be considered is the BMP signaling, which is the key driver of HBs, which was proved to be essential for the bipotency maintenance of HBs [6]. The absence of BMP signaling causes hepatic differentiation shifts to other lineages [26]. Although we encountered some difficulties due to the lack of reliable agonists in the BMP pathway, fortunately, we found that Vc could phosphorylate BMP downstream SMAD1/5/8, supporting the preservation of HBs identity. Vc was also reported to phosphorylate SMAD1/5/8 and potentiate BMP signaling pathway activity in cardiomyogenesis [10].

So far, chemical cocktail-derived HLCs were generally characterized by hepatic marker genes and protein expression, albumin secretion, and glycogen uptake [11, 24, 27], although several reports analyze hepatic CYP enzyme metabolic activities but showed poor functional activities in comparison to PHs [28, 29]. Therefore, it is necessary to optimize the chemical differentiation formula for the functional maturation of the induced HLCs. As an option, recent studies demonstrated that 3D culture could promote the metabolic maturation of differentiated HLCs [15, 16, 30, 31]. It has been reported that 3D hepatosphere culture increased the HPLCs differentiation efficiency (from 50 to 80%) and also enhanced functional maturation of derived HLCs [15]. However, this cell aggregation culture mostly relied on U-shaped or ultra-low attachment microwell (96-well), which is unsuitable for large-scale cell production. The cells’ viabilities are size-dependent in 3D aggregated culture, while it is difficult to form and maintain a moderate and uniform size of hepatic cell spheroids in mass-formed suspension culture [32], which was also observed in our studies (Figure S11). Another study reported that the inclusion of an intermediate proliferative HB stage in hepatic differentiation protocol led to a more functional mature of the HLCs at the final maturation stage [5].

In our present study, the proliferative HBs could be further differentiated into functional mature HLCs efficiently, with various representative hepatocyte markers and typical functional characteristics. Especially, the metabolic activities of CYP enzymes were comparable to PHs, and this is a hallmark of functional mature hepatocytes. These results suggested these HLCs are capable of used in bio-artificial liver systems and drug toxicity analyses. In addition, these hepatic cells could engraft and differentiate into hepatocytes after transplanted into NSI mice with acute liver failure, resulting in considerable liver repopulation and helped the acute liver failure mice to survival through. These results indicated that our chemical-based hepatic expansion and maturation method induces acquired hepatocytes to function in vivo and in vitro.

## Conclusions

In summary, we have developed a robust and cost-effective chemical strategy for the generation of functional hepatocytes. The key novelty is that a full chemical and defined culture condition supports HB expansion with preserved bipotency and enhances capacity for further hepatocyte differentiation. Additionally, our current method, compared with other reported protocols, showed advantages in the long-term maintenance of HBs stemness and is highly efficient in differentiating into mature hepatocytes. Therefore, this study could serve as a foundation for future studies aimed at defining culture conditions using GMP compatible products and provides a novel cost-effective way for generating large-scale and functional hepatocytes.

## Supplementary Information

**Additional file 1.**

## Data Availability

The datasets supporting the conclusions of this article are included within the article.

## Abbreviations

hPSCs: Human pluripotent stem cells
HBs: Hepatoblasts
HLCs: Hepatocyte-like cells
PH: Primary hepatocyte
NSI mice: NOD-SCID-IL2RG^−/−^ mice
DMN: Dimethylnitrosamine
CHIR: CHIR99021
CYP: Cytochrome P450
ICG: Indocyanine green
PAS: Periodic acid-Schiff staining
