# Supplementary material for: Robust expansion and functional maturation of human hepatoblasts by chemical strategy
Source: Stem Cell Res Ther. 2021 Feb 25;12:151. doi: 10.1186/s13287-021-02233-9 (PMC7908723; doi:10.1186/s13287-021-02233-9)
Supplement: Supplementary file 1 — Additional file 1. [file 13287_2021_2233_MOESM1_ESM.docx]

**Supporting information**

**Robust expansion and functional maturation**

**of human hepatoblasts by chemical strategy**

Tingcai Pan^1, 3, 4, †^, Jiawang Tao^1, 2, 3, †^, Yan Chen^1, 3^, Jiaye Zhang^1, 3,^, Anteneh Getachew^1, 2, 3^, Yuanqi Zhuang^1, 3^, Ning Wang^1, 3^, Yingying Xu^1, 3^, Shenglin Tan^1, 2, 3^, Ji Fang^1, 3^, Fan Yang^1, 3^, Xianhua Lin^1, 3^, Kai You^1, 3^, Yi Gao^4,^ * and Yin-xiong Li^1, 2, 3, 5,^ *

^1^ Institute of Public Health, Guangzhou Institutes of Biomedicine and Health (GIBH), Chinese Academy of Sciences, Guangzhou, 510530, China

^2^ University of Chinese Academy of Science, Beijing, 100049, China

^3^ Key Laboratory of Regenerative Biology, South China Institute for Stem Cell Biology and Regenerative Medicine, Guangdong Provincial Key Laboratory of Biocomputing, Guangzhou Institutes of Biomedicine and Health, Chinese Academy of Sciences, Guangzhou, 510530, China

^4^ Department of Hepatobiliary Surgery Ⅱ, Zhujiang Hospital, Southern Medical University, Guangzhou 510280, Guangdong Province, China

^5^ Bioland Laboratory (Guangzhou Regenerative Medicine and Health Guangdong Laboratory), 510005 Guangzhou, China

^†^ These authors contributed equally to this work.

* Correspondence: gaoyi6146@163.com and li_yinxiong_iph@gibh.ac.cn.

**Methods**

**1. Oil red O Staining**

The HLCs were fixed in 4% PFA (Sigma-Aldrich) for 30 min at room temperature, washed with PBS, and treated with 60% isopropanol for 1 min at room temperature. The HLCs were then incubated with 60% Oil red O (Muto Pure Chemicals, Tokyo, Japan) in water for 20 min at room temperature. After washing with 60% isopropanol for 1 min and additional washing with PBS, the cells were incubated with hematoxylin (Muto Pure Chemicals) for 5 min.

**2. Alexa-Flour 488-ac-LDL Staining**

The HLCs were cultured with medium containing Alexa-Flour 488-ac-LDL for 1 h, and immunohistochemistry was performed. Nuclei were counterstained with DAPI.

**Supplementary figures**


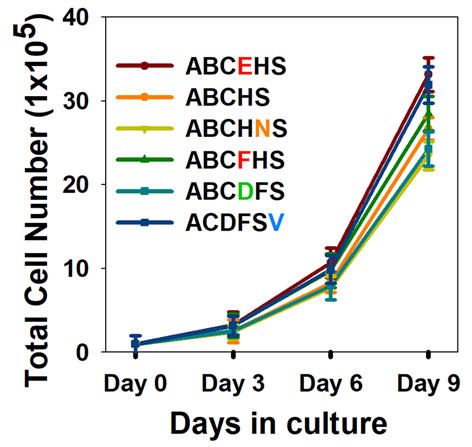


**Fig. S1. Growth curve of HBs cultured in different conditions.**

Cell growth curves were analyzed by obtaining a cell count. Data are presented as mean ± SEM, n = 3.


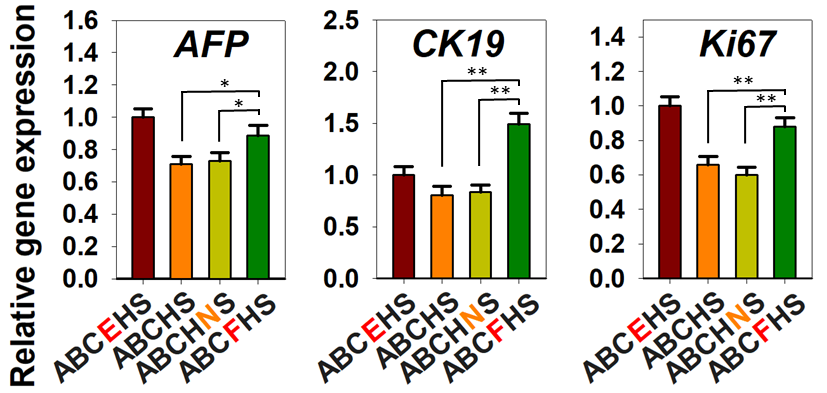


**Fig. S2. Gene expression in HBs treated with different chemical cocktails.**

RT-PCR results showing *AFP*, *CK19* and proliferative gene *Ki67* expression in HBs treated with different chemical cocktails. Data are presented as mean ± SEM, n = 3. * *P*< 0.05, ** *P*< 0.01.


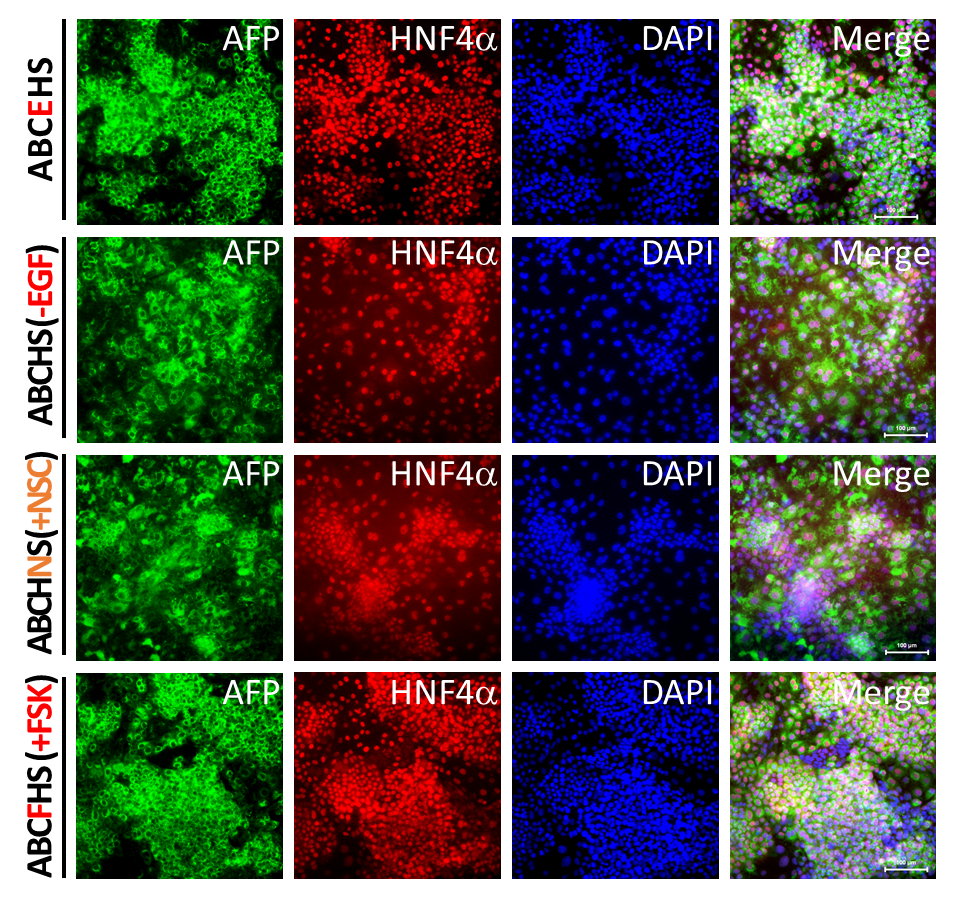


**Fig. S3. HB specific marker AFP and HNF4α expression after different chemical cocktails treated.**

Immunostaining analyses of HB specific marker AFP and HNF4α expression after different chemical cocktails treated. Scale bars 100 μm.


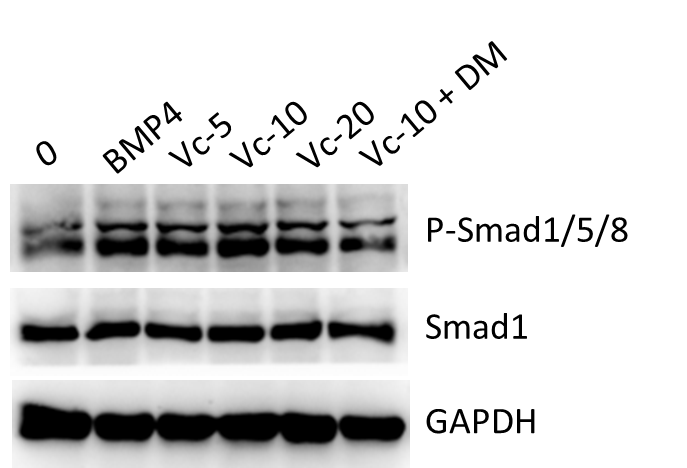


**Fig. S4. Vitamin C phosphorylate Smad1/5/8 in HBs culture.**

Western blot analyzed phosphorylation of Smad1/5/8 after Vc treated in HBs. Vc-5: 5 μg/mL Vc, Vc-10: 10 μg/mL Vc, Vc-20: 20 μg/mL Vc, DM: 0.5 μM Dorsomorphin.


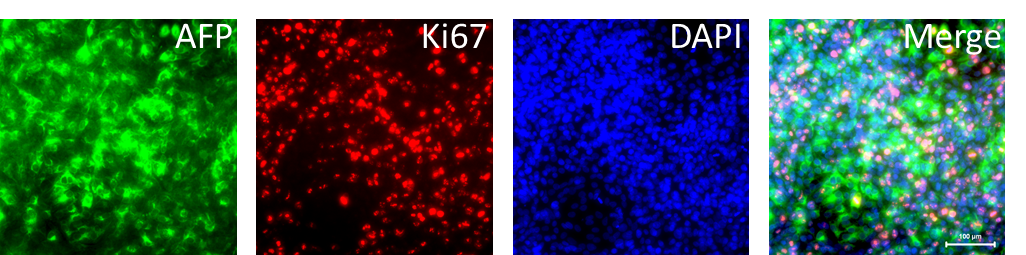


**Fig. S5. AFP and Ki67 expression on the early passage (passage 5) of the expanded HBs.**

Immunostaining analyses of AFP and Ki67 expression on the early passage (passage 5) of the expanded HBs. Scale bars 100 μm.


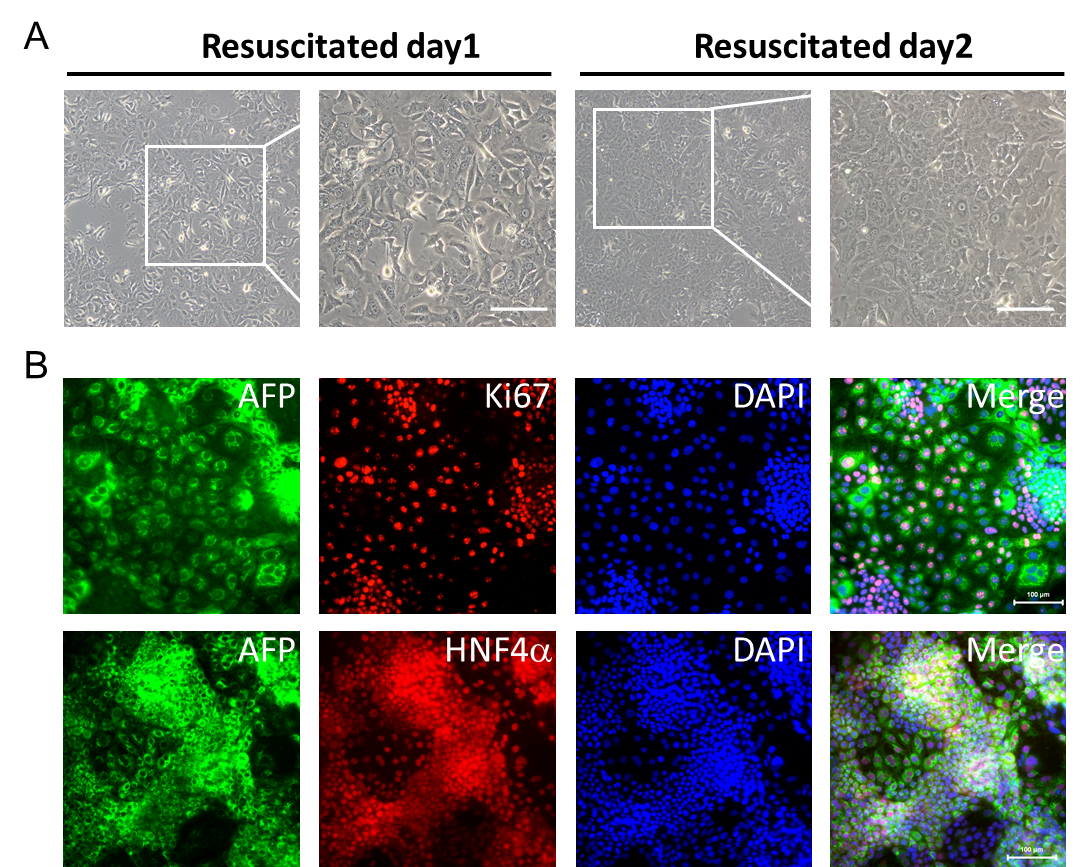


**Fig. S6. Characterization of the cryopreserved and resuscitated HBs.**

(A) The morphology of the resuscitated HBs. Scale bars 100 μm. (B) Immunostaining analyses of AFP, HNF4a and Ki67 expression on the cryopreserved (at passage 10) and resuscitated HBs.


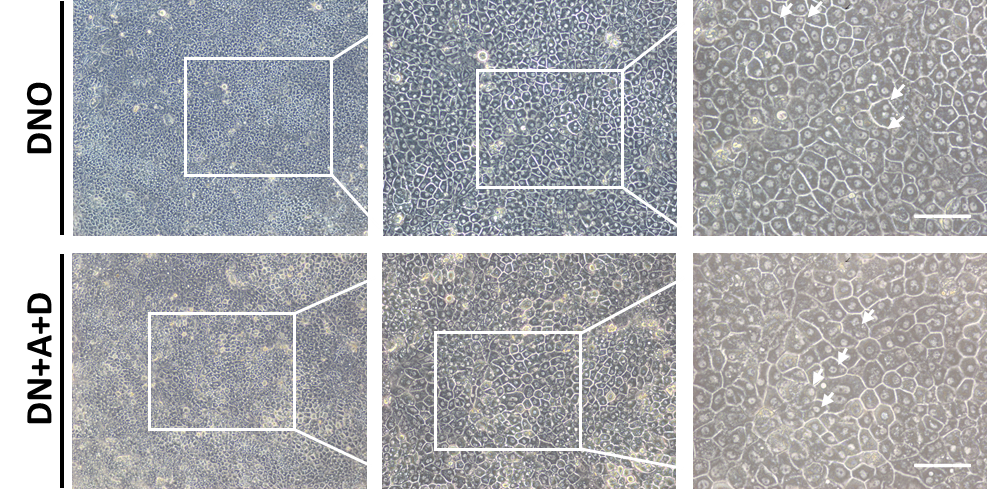


**Fig. S7. The morphology of expanded HBs derived HLCs.**

After 6 days of induction with hepatic maturation condition, the expanded HBs efficiently differentiated into a homogenous HLCs with typical hepatocyte morphology, with polygonal shape and distinct round nuclei even some has two nuclei (White arrows). Scale bar 100 μM.


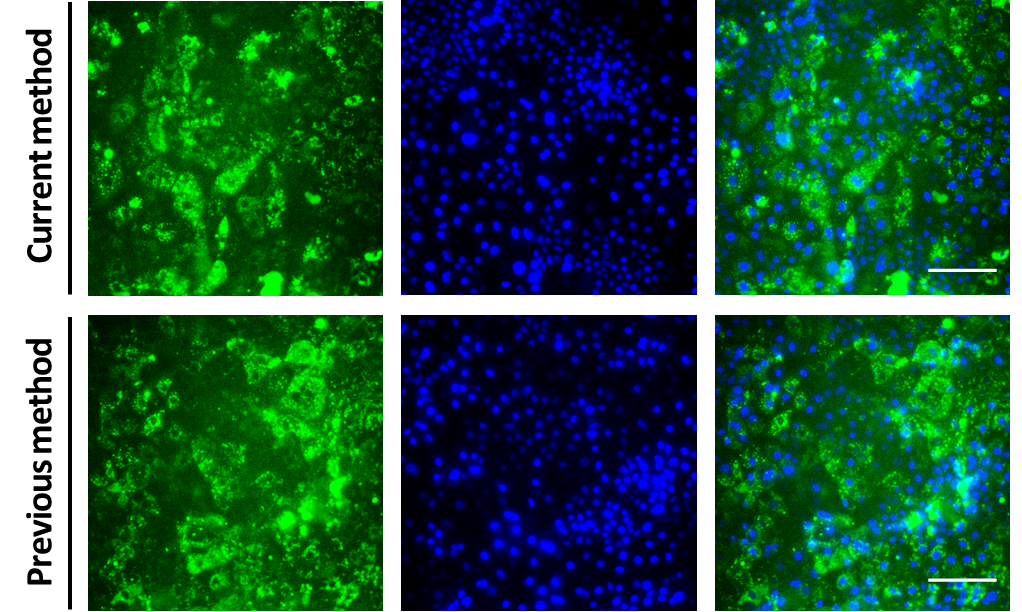


**Fig. S8. The HLCs were stained by Alexa-Flour 488-ac-LDL.**

The HLCs were cultured with medium containing Alexa-Flour 488-ac-LDL (green) for 1 h, and immunohistochemistry was performed. Nuclei were counterstained with DAPI (blue). Scale bar 100 μM.


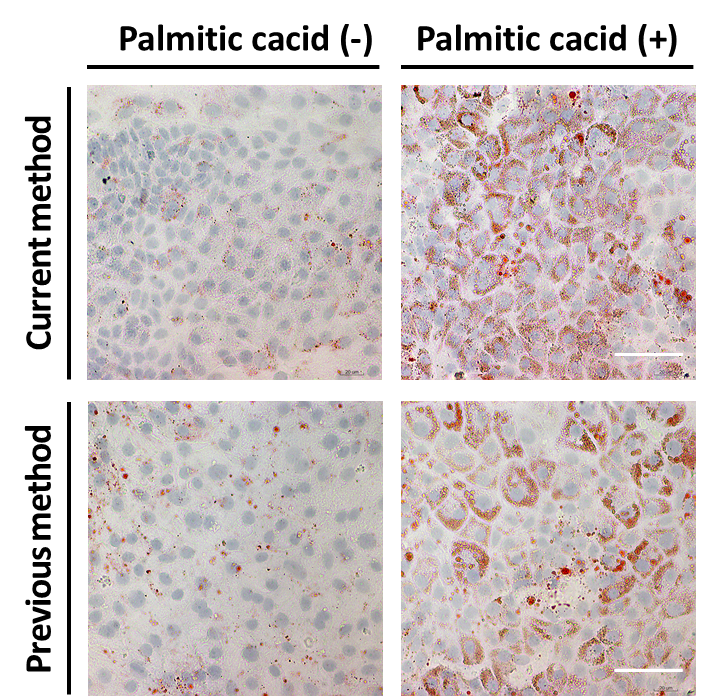


**Fig. S9. The HLCs can synthesize lipids from a saturated fatty acid.**

Oil red O staining was conducted for derived HLCs cultured with palmitic acid, a saturated fatty acid, and without any fatty acids (as control). DNA was stained with hematoxylin. Scale bars, 100μm.


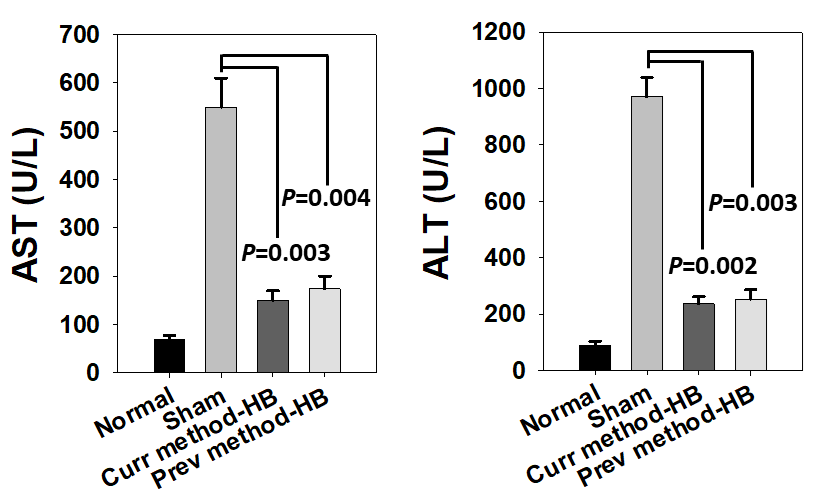


**Fig. S10. Detection of ALT and AST levels in mice serum.**

Detection of ALT and AST levels in mice serum at one week after HBs transplantation. Data analyzed by 2-tailed t tests.


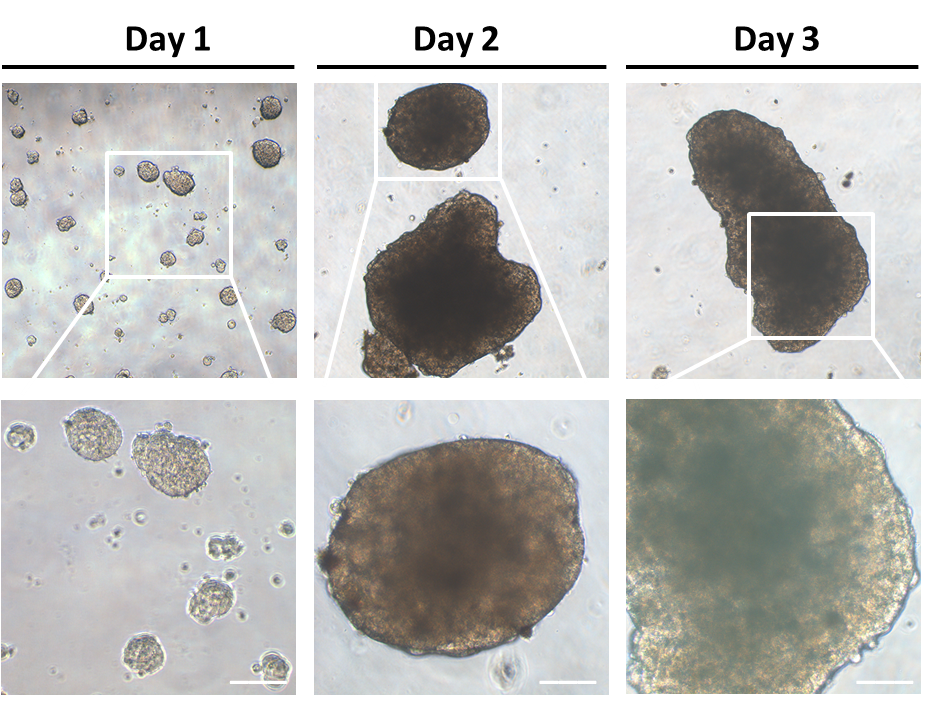


**Fig. S11. Heterogeneous size of HBs spheroid in suspension culture.**

HBs suspension culture in low-attachment wells produced spheroid population with heterogeneous and uncontrollable size. Scale bar 200 μM.

**Supplementary Tables**

**Table 1. List of antibodies used in this study**

| **Primary anbibody** | **Species** | **Company** | **Cat Number** | **Dilution** |
| --- | --- | --- | --- | --- |
| HNF4α | Rabbit | Genetex | GTX62347 | 1:200 |
| AFP | Mouse | Genetex | GTX84948 | 1:200 |
| E-CAD | Goat | R&D systems | AF648 | 1:200 |
| Ki67 | Rat | Thermo Fisher | 11-5698-82 | 1:100 |
|  | Rabbit | Genetex | GTX16667 | 1:200 |
| Ep-CAM | Mouse | Invitrogen | 53-8326-42 | 1:100 |
|  | Mouse | Abcam | ab46714 | 1:100 |
| ALB | Goat | R&D systems | MAB1455 | 1:200 |
|  | Rabbit | Dako | F0117 | 1:50 |
| SOX9 | Mouse | Abcam | ab76997 | 1:200 |
| OCT4 | Mouse | Genetex | GTX627423 | 1:200 |
| AAT | Rabbit | Abcam | AB166610 | 1:200 |
| CYP3A4 | Mouse | Genetex | GTX60577 | 1:200 |
| CYP2C9 | Rabbit | Genetex | GTX55583 | 1:200 |
|  | | | | |
| **Second antibody** | **Species** | **Company** | **Cat. Number** | **Dilution** |
| Anti-Goat IgG Antibody, Alexa Fluor 488 | Donkey | Invitrogen | A-11055 | 1:500 |
| Anti-Goat IgG Antibody, Alexa Fluor 568 | Donkey | Invitrogen | A-11057 | 1:500 |
| Anti-Mouse IgG Antibody, Alexa Fluor 488 | Donkey | Invitrogen | A32766 | 1:500 |
| Anti-Mouse IgG Antibody, Alexa Fluor 568 | Donkey | Invitrogen | A10037 | 1:500 |
| Anti-Rabbit IgG antibody Alexa Fluor 488 | Donkey | Invitrogen | A-21206 | 1:500 |
| Anti-Rabbit IgG antibody Alexa Fluor 568 | Donkey | Invitrogen | A10042 | 1:500 |

**Table 2. List of primers for real-time PCR**

| **Gene** | **Primer sequence (forward / reverse; 5' to 3')** |
| --- | --- |
| *AFP* | AGAACCTGTCACAAGCTGTG / GACAGCAAGCTGAGGATGTC |
| *HNF4α* | TGTACTCCTGCAGATTTAGCC / CTGTCCTCATAGCTTGACCT |
| *CK19* | ACCAAAGCTCACGCGTGGAAA / TGATGTGTCTCTCGGTCAAGTT |
| *Ki67* | GCCTGCTCGACCCTACAGA / GCTTGTCAACTGCGGTTGC |
| *ALB* | TGCAACTCTTCGTGAAACCTATG / ACATCAACCTCTGGTCTCACC |
| *AAT* | ATGCTGCCCAGAAGACAGATA / CTGAAGGCGAACTCAGCCA |
| *ARG1* | TGGACAGACTAGGAATTGGCA / CCAGTCCGTCAACATCAAAACT |
| *ASS1* | AGGAAAGGGGAACGATCAGGT / GTGTTGCTTTGCGTACTCCA |
| *ASL* | CAGTGGACCCCATCATGGAGA / GGCTTTGCTGCCTTGAACATC |
| *CPS1* | ACTTCAGTTGAGTCCATTATGGC / GGAACGGATCATCACTGGGTAG |
| *CYP2D6* | GTGTCCAACAGGAGATCGACG / CACCTCATGAATCACGGCAGT |
| *CYP3A4* | TTCAGCAAGAAGAACAAGGACAA / GGTTGAAGAAGTCCTCCTAAGC |
| *CYP2C9* | CTACAGATAGGTATTAAGGACA / GCTTCATATCCATGCAGCACCAC |
| *CYP1A2* | CTTCGTAAACCAGTGGCAGG / AGGGCTTGTTAATGGCAGTG |
| *GAPDH* | CAAAGTTGTCATGGATGACC / CCATGGAGAAGGCTGGGG |
